# Centering Local Knowledge to Address the Imbrication of Settler Colonialism and Global Health: Comment on "The Rhetoric of Decolonizing Global Health Fails to Address the Reality of Settler Colonialism: Gaza as a Case in Point"

**DOI:** 10.34172/ijhpm.8698

**Published:** 2024-12-15

**Authors:** Megan K. Muller

**Affiliations:** Department of Anthropology, University of British Columbia, Vancouver, BC, Canada.

**Keywords:** Settler Colonialism, Decolonization, Global Health, Gaza, Indigenous Health

## Abstract

In the article "The Rhetoric of Decolonizing Global Health Fails to Address the Reality of Settler Colonialism: Gaza as a Case in Point," Engebretsen and Baker call on researchers to re-examine the ways we employ the rhetoric of decolonization in global health. They critique the "reformist" strand of decolonization which fails to mitigate structural inequities resulting from settler colonialism. I extend the authors’ work by considering how researchers might harness decolonial approaches to identify and nuance the ways power relations, on a regional, national, or global level, lead to unnecessary suffering. I assert that this requires centering Indigenous voices and local knowledge and de-centering Eurocentric frameworks and presumed universality. My hope is that by being precise with the language we use to denounce atrocities, this will engender commitments and accountabilities that determine whether the response coming from global health leaders moves us towards increased health equity rather than empty rhetoric.

 In the recent article “The Rhetoric of Decolonizing Global Health Fails to Address the Reality of Settler Colonialism: Gaza as a Case in Point,” Engebretsen and Baker call attention to how researchers might respond to the devastating humanitarian crisis wrought by recent Israeli air strikes targeting health institutions in Gaza. The authors call on researchers to re-examine the ways we employ the rhetoric of decolonization in global health. They critique the “reformist” strand of decolonization which fails to mitigate structural inequities by focusing on promoting surface level “diversity.” Identifying Gaza as a case in point, the authors criticize global health institutions, such as the World Health Organization (WHO), for doing little more for Palestinian victims beyond calling for a ceasefire. Importantly, the authors assert that:

 “*The apparent powerlessness of these institutions in the face of overwhelming violence and obstruction by powerful governments highlights the necessity for global health to address the nature of settler colonialism in the 21st century and its own imbrication within the structures that sustain these colonial practices.*”^1^

 Addressing the nature of settler colonialism is a fundamentally important goal if we are to take seriously the international commitments to reduce the health disparities faced by Indigenous populations.^2^ Yet, settler colonialism is a complex, multifaceted, and pervasive socio-political and economic phenomenon. Doing so will further necessitate reflecting on questions such as: How might we go about studying the imbrication of settler colonialism and global health and what knowledge sources and interpretive paradigms are privileged in the process? How do we account for the ways global power relations take on different meanings in diverse localities? I wish to extend Engebretsen and Baker’s work by considering the implications of their call to decolonize global health by “ground[ing] knowledge creation in grassroots realities,”^1^ by drawing on interdisciplinary perspectives on health and settler colonialism. To begin, I feel is it necessary to situate myself as a descendant of European settlers, a non-Indigenous ally, and a proponent of decolonizing healthcare systems in Canada (where I live and work) and abroad.

 Colonialism has and continues to be a driving force of the global spread of capitalism over the past few centuries. Settler colonialism is conceived as a process rather than a historical event, through the ongoing acquisition of territory.^3^ The means of acquiring territory have taken diverse historical forms across the globe, yet, in all cases, the imperative to possess land and resources for economic development continues to take precedence over Indigenous claims to territory, self-governance, and even basic human rights. In a seminal article, historian Patrick Wolfe explains the logic of elimination as an “organizing principle of settler-colonial society.”^4^ The acquisition and occupation of new territories requires the elimination of the original inhabitants, or their existence as a uniquely identifiable group, and their claim to sovereignty. While settler colonialism has taken up specific socio-political configurations in different social contexts and historical moments, in the case of Gaza, settler colonialism is unfolding in its most deadliest of forms.

 In Wolfe’s formulation, genocidal violence is but one of several possible manifestations of the logic of elimination. Wolfe claims that the difference between genocide, as a violent political tactic, and settler colonialism, as a broader political structure, is that settler colonialism is relatively impervious to regime change. The tactics employed to support settler colonialism can transmute “into different modalities, discourses and institutional formations as it undergirds the historical development and complexification of settler society.”^4^ Other modalities of settler colonialism include cultural assimilation, or targeted government policies aimed at absorbing minority groups into the dominant groups norms, beliefs and legal and economic system,^5^ and liberal recognition, a legal framework through which marginalized groups assert their claims to political and cultural self-determination.^6^ While the recognition modality appears as an improvement to the more violent and coercive tactics, Indigenous scholars have critiqued the liberal recognition paradigm, questioning whether it is possible to forge equitable relationships between the colonial governments and the Indigenous peoples who have been forcefully incorporated or displaced by the settler state.^7,8^ We may thus understand the logic of elimination intrinsic to settler colonialism as existing on a continuum that oscillates between the more covert processes aimed at eradicating Indigenous cultures and the eruptive “genocidal moments”^9^ of mass violence and destruction, such as is occurring in Gaza. A contextualized understanding of how and why settler colonialism takes on particular modalities in specific historical moments may prove critical to identifying and addressing colonial violence.

 Engebretsen and Baker claim that decolonization rhetoric, as a response to the injustices and violence resulting from settler colonialism, have ultimately failed “to address the enduring violence and oppression perpetuated by the global political economy, which is underpinned by colonial and capitalist ideologies.”^1^ While the authors’ call to recognize how “globally enmeshed relations” perpetuate the exploitative capitalism and displacement from ancestral lands, I would add that this also requires a sensitivity to specific historical and socio-political conditions. In some cases, seemingly benevolent medical humanitarianism can be experienced as a kind of violence, in the same way that the reluctance of global health institutions to intervene and provide services and resources can also perpetuate health inequity. Notable examples of humanitarian interventions that caused lasting trauma in the Canadian context include the quarantining of tuberculosis-positive Inuit in southern sanitaria^10^ segregated Indian hospitals,^11^ and forced sterilization.^12^ Internationally, medical anthropologists have explored how dominant western liberal philosophy articulates an ethics which justifies the refusal of care to groups existing outside of the dominant frameworks for recognition and rights.^13-15^ Thus, the impact of global health interventions takes on nuanced meanings in various localities. By highlighting these complexities, my aim is to advocate for research that explores contextual contingencies by centering local knowledge, as fundamental to envisioning equitable interventions and global health mandates. I believe the creation of contextually-informed knowledge on the links between settler colonialism and health is akin to what the authors may consider an alternative to the dominant “top-down knowledge translation” paradigm.

 Engebretsen and Baker’s critique of the presumed universality of health knowledge generated in the global north is an important intervention, as the co-optation of decolonization rhetoric can work to further mask manifestations of colonialism as a root cause of health disparity. Indeed, the deployment of discourse is a key tactic of modern governance.^16^ Seemingly progressive discourse often takes on disparate meanings when studied from a grassroots perspective. To provide an example, in the context of Canadian North Pacific coast, the designation of “rural/remote” locality by health institutions normalizes substandard access to health services in Indigenous communities. Yet, from the perspective of community members, the discourse of “rural/remote” is part of a colonial imaginary about centre and periphery of colonized territories, contradicting Indigenous views of territory/home as central.^17^ In the context of emergency medical departments, a widely circulated discourse of egalitarianism, or that all patients are treated the same, masks how healthcare is differentially accessed by populations that are subject to medical racism and/or face significant barriers in access to health services.^18,19^ These small scale examples demonstrate some of the complex ways a logic of elimination undergirding settler colonialism is obscured and the health disparities faced by colonized peoples are normalized. Returning to the case of Gaza, Amal Jamal, a Palestinian political scientist, discusses how the Israeli regime has been analyzed via a western democratic tradition with a focus on “modernizing” supposedly backwards peoples. While this is shared across the colonial experience, Jamal builds on the work of Elia Zureik, to explain that “Palestinian physical presence is framed as a security challenge.”^20^ This adds another layer of complexity, as military action and resulting violence are framed as a measure to *prevent* violence. In this configuration, a response from global health institutions requires contending with the discursive problematic of a right to national security.

 What I found was lacking from Engebretsen and Baker’s article was further analysis directed towards the link between the substantial decolonization in health research that they call for, and the possibility to end the genocide inflicted upon Palestinians living in Gaza. The authors’ suggest that a reluctance from the WHO to intervene should be framed not as apolitical neutrality, but rather as a deliberate inaction that implicitly supports colonial genocide. While I am in agreement with Engebretsen and Baker that the conceptualization of global health as an apolitical endeavor is a fallacy, I provide the previous examples to encourage researchers and health leaders to consider the nuanced ways settler colonialism is perpetuated by global political powers in different localities, as this requires distinct strategies and political resources to confront them.

 My concluding thought is that, while I appreciate the authors effort at re-centering settler colonialism as the focus of decolonization efforts, I am left wondering if settler colonialism is perhaps too broad a concept on its own to achieve “confronting material colonial infrastructures”^1^ with an action-to-knowledge strategy. Drawing upon the theorizations set forth by Indigenous scholars, we can harness research to identify and nuance the ways power relations, on a regional, national, or global level, lead to unnecessary suffering. Settler colonialism, as a conceptual framing, allows us to recognize structural inequities and racist legacies, yet it alone does not allow for an analysis of how different strategies are employed in different socio-political contexts to support the underlying logic of elimination. My concern is that broad characterizations, as an almost faceless, insidious force, can further engender political polarization where what is needed is opportunities for actionable steps to remedy conflict and restore the health of colonized populations. My aim as a non-Indigenous ally is to bring attention to these issues and to advocate for a fair redistribution of land and political power to colonized peoples. I assert that this requires, firstly centering Indigenous voices, and secondly, de-centering Eurocentric frameworks and presumed universality by creating space in global health research paradigms for grassroots understandings of settler colonialism. My hope is that by being deliberate and precise with the language we use to denounce atrocities this will engender commitments and accountabilities that determine whether the response coming from global health leaders moves us towards increased health equity rather than empty rhetoric.

## Ethical issues

 Not applicable.

## Conflicts of interest

 Author declares that she has no conflicts of interest.
